# Obituary

**Published:** 1858-11

**Authors:** 


### OBITUARY.

Died, in this city, on the 6th November, Dr. GEORGE BARTLETT FOSTER, at the age of thirty-three years. Dr. FOSTER was a native of Roxbury, Mass., and was the grandson of Dr. George Bartlett, a distinguished practitioner of that city some years since, and still well remembered by many now living. The deceased was graduated at the University of Vermont, and received his medical degree from the Jefferson Medical College, Philadelphia, in the spring of 1854. With the exception of the time given to a course of lectures at Philadelphia, he pursued his medical studies in Boston, having had the advantage of a year's stay at the city hospital at Deer Island, as resident pupil. After his graduation at Philadelphia, he was for three months acting assistant-physician at the State hospital at Rainford Island; and in the fall of '54 visited Paris, to continue his studies under the distinguished medical teachers of that city. Soon after his return to this country in the fall of '55, he came to this city, to establish himself in the practice of his profession, to which he devoted himself with energy and uniform good success, till prevented by the illness which immediately preceded his death. His last illness was of a week's duration.

His disease was purpura hæmorrhagica, complicated with enlarged liver and jaundice. All the symptoms were of marked severity, and the prognosis was from the beginning unfavorable. The attack was ushered in by neuralgic pains and a sense of depression and malaise. The hæmorrhage from the mucous surfaces was very great, and the secretion of bile was not re-established. There was marked prostration throubgout, and the delirium which was present at first soon passed into stupor.

Dr. FOSTER was a man of fine qualities of heart; an ardent and unwavering friend. Generous and impulsive, he was ever ready to encourage and assist those who sought his advice and aid. Though sensitive to the opinions of others, he had much force and energy of character, and was not easily disheartened or deterred from carrying out his resolves or convictions.

In his profession he was earnest, diligent and for the short time that he was engaged in practice, remarkably successful. He had the advantage of a thorough medical education, and an opportunity to observe the practice of many of the men most eminent in this country and France, for their medical attainments. Added to this, he had those mental endowments which, with experience and study, are sufficient to insure an honorable rank in his profession. His manners at the bedside of the sick, were more than ordinarily winning, and there are many who gratefully remember the encouragement and confidence which he inspired. He never refused his services, when in health, to those who called for them, and never shrank from doing, cheerfully, his full share of that gratuitous labor which the members of the medical profession are so familiar with and generally so willingly undertake.

